# Causes of Stranding and Mortality, and Final Disposition of Loggerhead Sea Turtles (*Caretta caretta*) Admitted to a Wildlife Rehabilitation Center in Gran Canaria Island, Spain (1998-2014): A Long-Term Retrospective Study

**DOI:** 10.1371/journal.pone.0149398

**Published:** 2016-02-22

**Authors:** Jorge Orós, Natalia Montesdeoca, María Camacho, Alberto Arencibia, Pascual Calabuig

**Affiliations:** 1 Department of Morphology, Veterinary Faculty, University of Las Palmas de Gran Canaria, Arucas (Las Palmas), Spain; 2 Department of Animal Pathology, Veterinary Faculty, University of Las Palmas de Gran Canaria, Arucas (Las Palmas), Spain; 3 Department of Clinical Sciences, Veterinary Faculty, University of Las Palmas de Gran Canaria, Arucas (Las Palmas), Spain; 4 Tafira Wildlife Rehabilitation Center, Cabildo de Gran Canaria, Tafira Baja-Las Palmas de Gran Canaria, Spain; Faculty of Animal Sciences and Food Engineering, University of São Paulo, BRAZIL

## Abstract

**Aims:**

The aims of this study were to analyze the causes of stranding of 1,860 loggerhead turtles (*Caretta caretta*) admitted at the Tafira Wildlife Rehabilitation Center in Gran Canaria Island, Spain, from 1998 to 2014, and to analyze the outcomes of the rehabilitation process to allow meaningful auditing of its quality.

**Methods:**

Primary causes of morbidity were classified into seven categories: entanglement in fishing gear and/or plastics, ingestion of hooks and monofilament lines, trauma, infectious disease, crude oil, other causes, and unknown/undetermined. Final dispositions were calculated as euthanasia (E_r_), unassisted mortality (M_r_), and release (R_r_) rates. Time to death (T_d_) for euthanized and dead turtles, and length of stay for released (T_r_) turtles were evaluated.

**Results:**

The most frequent causes of morbidity were entanglement in fishing gear and/or plastics (50.81%), unknown/undetermined (20.37%), and ingestion of hooks (11.88%). The final disposition of the 1,634 loggerhead turtles admitted alive were: E_r_ = 3.37%, M_r_ = 10.34%, and R_r_ = 86.29%. E_r_ was significantly higher in the trauma category (18.67%) compared to the other causes of admission. The highest M_r_ was observed for turtles admitted due to trauma (30.67%). The highest R_r_ was observed in the crude oil (93.87%) and entanglement (92.38%) categories. The median T_r_ ranged from 12 days (unknown) to 70 days (trauma).

**Conclusions:**

This survey is the first large-scale epidemiological study on causes of stranding and mortality of Eastern Atlantic loggerheads and demonstrates that at least 71.72% of turtles stranded due to anthropogenic causes. The high R_r_ (86.29%) emphasizes the importance of marine rehabilitation centers for conservation purposes. The stratified analysis by causes of admission of the three final disposition rates, and the parameters T_d_ and T_r_ should be included in the outcome research of the rehabilitation process of sea turtles in order to allow comparative studies between marine rehabilitation centers around the world.

## Introduction

Two families and seven species of sea turtles are currently recognized [1], all of which are included in the Red List of the World Conservation Union [2]. The family Cheloniidae includes the green turtle (*Chelonia mydas*), loggerhead (*Caretta caretta*), hawksbill (*Eretmochelys imbricata*), Kemp’s ridley (*Lepidochelys kempi*), olive ridley (*Lepidochelys olivacea*), and flatback turtle (*Natator depressus*). The family Dermochelyidae includes only the leatherback (*Dermochelys coriacea*) [1]. The most common species around the Canary Islands is the loggerhead turtle, mainly coming from the US western Atlantic by the Gulf Stream [3].

There are reports of disease surveys of free-living sea turtles in Australia [4–9], Hawaii [10–16], Florida [17–26], Brazil [27], France [28], Italy [29], and the Canary Islands [30]. However, long-term epidemiological studies of sea turtle diseases covering more than one decade are scarce [14–16,21,29], and only rarely the survival rates have been thoroughly analyzed [14].

The aims of this study were to analyze the causes of stranding in a large population of loggerhead turtles admitted to the Tafira Wildlife Rehabilitation Center (TWRC) in Gran Canaria Island, Spain, from 1998 to 2014 using specific epidemiological data, to compare these results with those obtained in other geographic regions, and to analyze the outcomes of the rehabilitation process to allow meaningful auditing of its quality.

## Materials and Methods

### Ethics Statement

Sea turtle rehabilitation program at the TWRC was conducted with authorization of the Wildlife Department of the Canary Islands Government (Ms. Guacimara Medina), and the Environment Department of the Cabildo de Gran Canaria (Ms. María del Mar Arévalo). Animal work and all sampling procedures were specifically approved by the TWRC Animal Care Committee and the insular government Cabildo de Gran Canaria, and were consistent with standard vertebrate protocols and veterinary practices. Loggerhead turtles that had to be euthanized for animal welfare reasons were administered barbiturates by intravenous injection.

### Animals and study area

A retrospective study was performed using the original medical records of 1,860 loggerhead turtles admitted to the TWRC, Gran Canaria Island, Spain, from 1998 to 2014. The TWRC receives turtles stranded in Gran Canaria and eventually from other islands of the Canary Islands archipelago. Gran Canaria (27°73’-28°18’N and 15°35’-15°83’W) is the third largest island (1,560.1 km^2^) of the Canary Islands archipelago and has a coastline of 236 km.

### Variables analyzed

Straight carapace length (SCL), weight, stranding point, date and primary cause of admission, and final disposition including date of death or release were recorded. Sex was only determined in 248 turtles by gonadal examination at necropsy. No complete records of SCL and weight were found for 565 turtles. Turtles were categorized as pelagic juveniles (SCL <42 cm), juveniles-subadults (42 cm ≤ SCL <70 cm) and adults (SCL ≥70 cm) according to previous studies [31–33]. In order to study the seasonality of the different causes of admission, the year was divided into four seasons: spring (from March to May), summer (June to August), fall (September to November) and winter (December to February).

We defined the primary cause of morbidity as the main condition responsible for the turtle’s need for treatment [30]. When several causes were observed in the same turtle, clinical history and complementary studies were crucial to determine the primary cause of morbidity, and only this primary cause was recorded. Primary causes of morbidity were classified into seven categories: entanglement in derelict fishing gear and/or plastics (synthetic raffia), ingestion of hooks and monofilament lines, trauma (boat strikes), infectious disease, crude oil, other causes, and unknown/undetermined. The infectious disease category was applied when a pathogenic microorganism was confirmed by microbiological or histopathological diagnosis. Other causes were subdivided into: ingestion of plastics, buoyancy disorders, shark attack, malnutrition, and miscellany. The malnutrition category comprised turtles suffering from cachexia in absence of other lesions.

To assign these categories we used different sources: (a) the physical examination performed by the veterinarian at the admission instance; (b) the information from the people that recovered the turtle, (c) the case history; and when possible (d) from complementary studies as radiology, hematology, blood chemistry, cytology, microbiology, parasitology, gross pathology, histopathology, and toxicology.

Three categories were established for studying the final disposition of the loggerheads admitted alive: euthanized turtles (based on poor quality of life and/or prognosis for survival in the wild), dead turtles during the hospitalization period, and released turtles into the wild. Thus, three percentage rates were calculated for the total of loggerheads admitted alive: euthanasia rate (E_r_), unassisted mortality rate (M_r_), and release (survival) rate (R_r_). In addition, these percentage rates were also calculated for each cause of admission.

The parameters time until death (T_d_; difference between the date of admission and the date of the death) for euthanized and dead turtles during the hospitalization period, and length of stay in the center for released turtles (T_r_; difference between the date of admission and the release date) were also evaluated for each cause of admission. Percentiles 10 (P_10_), 50 (P_50_), 75 (P_75_) and 90 (P_90_) for the variables T_d_ and T_r_ were also calculated.

### Statistical analysis

Statistical analyses were conducted using SPSS v.22.0 (SPSS Inc., Chicago IL) and R package v.3.1.0 (R Development Core Team 2014, Viena, Austria). Chi-square test (χ2) or Fisher exact tests were used to determine whether there was a significant difference between proportions. Odds Ratio (OR) measure of association was employed for disease comparisons. In order to study differences among years, trend analyses were applied for specific causes.

## Results

### Descriptive analyses

A total of 1,860 loggerhead turtles were included in this study. Most turtles (87.85%, n = 1,634) were alive when admitted. In the group of turtles whose SCL and weight were measured, the mean ± SD of the SCL and weight were 36.11±11.18 cm (range, 13.00–85.20 cm) and 9.33±8.21 kg (range, 0.27–55.5 kg), respectively. Thus, 69.34% (n = 898) of these turtles were classified as pelagic juveniles, 30.27% (n = 392) as juveniles-subadults, and only 0.39% (n = 5) as adults. 86.66% of turtles (n = 1,612) were classified as undetermined gender, 11.66% (n = 217) were sexed as females and 1.66% (n = 31) as males. In the group of turtles whose sex was determined the sex ratio was female-biased (7/1).

### Distribution of causes of morbidity

Number of cases and frequency distribution by causes of admission are shown in Table 1. The most frequent causes of morbidity were entanglement in fishing gear and/or plastics (50.81%, n = 945), unknown/undetermined (20.37%, n = 379), and ingestion of hooks and monofilament lines (11.88%, n = 221). The other primary causes had frequencies below 6%.

**Table 1:** Number of cases and frequency distribution by causes of admission in loggerhead turtles with different straight carapace length (SCL) stranded during the period 1998–2014.

Cause of admission	Number of cases					
	SCL < 42 cm	SCL ≥ 42–70 cm	SCL ≥ 70 cm	Unknown SCL	TOTAL	(%)
**Entanglement**	560	131	0	254	945	50.8
**Hooks/monofilament lines**	28	141	1	51	221	11.9
**Trauma (boat strike)**	35	35	1	26	97	5.2
**Infectious disease**	70	13	0	20	103	5.5
**Crude oil**	31	4	0	17	52	2.8
**Other causes**	37	10	1	15	63	3.4
Ingestion of plastics	7	4	0	8	19	1
Buoyancy disorder	3	3	0	0	6	0.3
Shark attack	3	1	0	1	5	0.3
Malnutrition	17	0	0	7	24	1.3
Miscellany	7	2	0	0	9	0.5
**Unknown/undetermined**	137	58	2	182	379	20.4
**TOTAL**	898	392	5	565	1,860	100

No differences between genders related to any of the analyzed causes were observed. Pelagic juvenile turtles (SCL < 42 cm) had a significant higher risk of entanglement (OR = 3.3; 95%CI: 2.6–4.3; *P* <0.0001), infectious disease (OR = 2.4; 95%CI: 1.3–4.5; *P* = 0.02) and crude oil (OR = 3.5; 95%CI: 1.2–10; *P* = 0.012) compared to juvenile/subadult and adult turtles. Conversely, the ≥42–70 cm SCL group had a significant higher risk of ingestion of hooks and monofilament lines (OR = 16.9; 95%CI: 11.1-25-8; *P* <0.0001) and trauma (OR = 2.3; 95%CI: 1.4–3.8; *P* <0.0001) compared to the other groups.

### Seasonality

Admissions were distributed as follows: 38.33% (n = 713) in summer, 27.20% (n = 506) in spring, 21.77% (n = 405) in fall, and 12.69% (n = 236) in winter.

**Fig 1:**
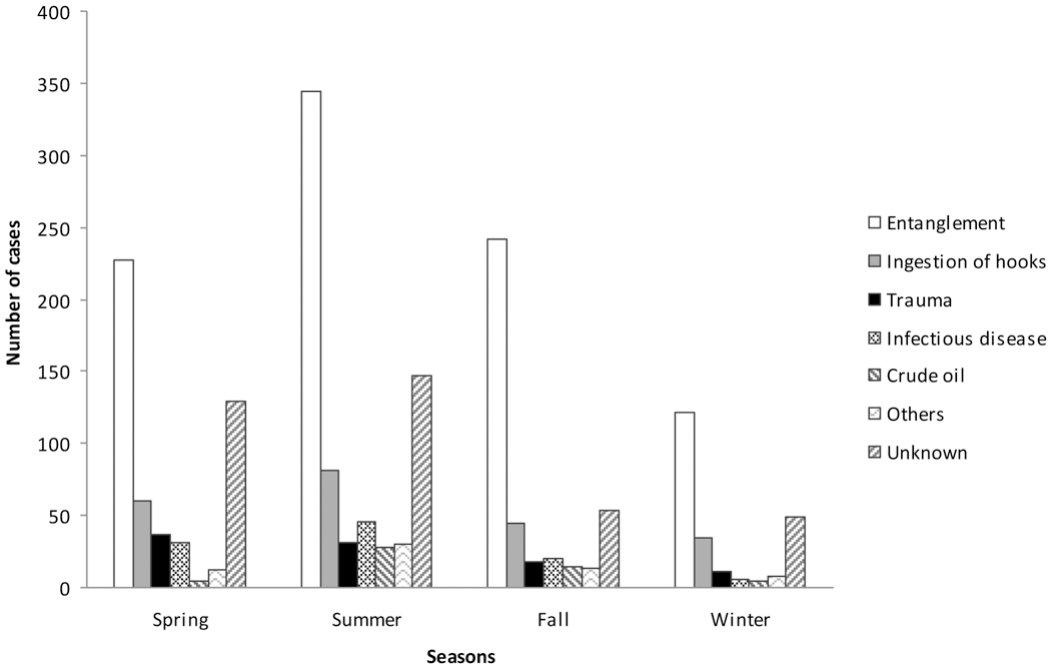
Seasonal variations in causes of admission during 1998–2014.

Seasonal variation in causes of admission is shown in Fig 1. Entanglement occurred quite frequently during all seasons (spring, 24.27%, n = 227; summer, 36.79%, n = 344; fall, 25.88%, n = 242; winter, 13.04%, n = 122), but it was significantly more prevalent in summer and fall (χ2 = 105.91, *P* < 0.0001). A significantly higher number of crude oil cases were observed in summer and fall (χ2 = 27.23, *P* < 0.0001). Ingestion of hooks, trauma, infectious disease, and unknown causes were more prevalent in spring and summer as compared to fall and winter (χ2 = 21.73, *P* < 0.0001; χ2 = 17.43, *P* = 0.001; χ2 = 33.42, *P* < 0.0001; χ2 = 81.89, *P* < 0.0001; respectively). We detected significant differences between seasons when other causes category was analyzed (χ2 = 18.07, *P* = 0.0001), being more prevalent in summer (47.61%, n = 30).

### Annual variation of causes of morbidity

**Fig 2:**
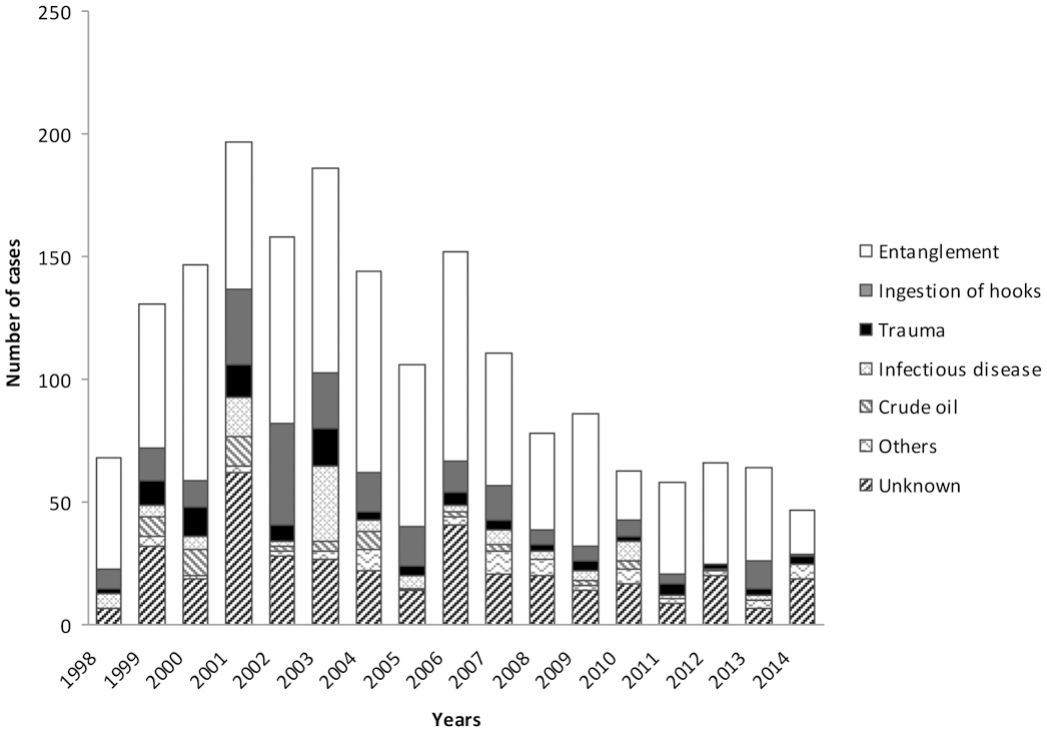
Annual variation in causes of admission of loggerhead turtles among the period 1998–2014.

**Fig 3:**
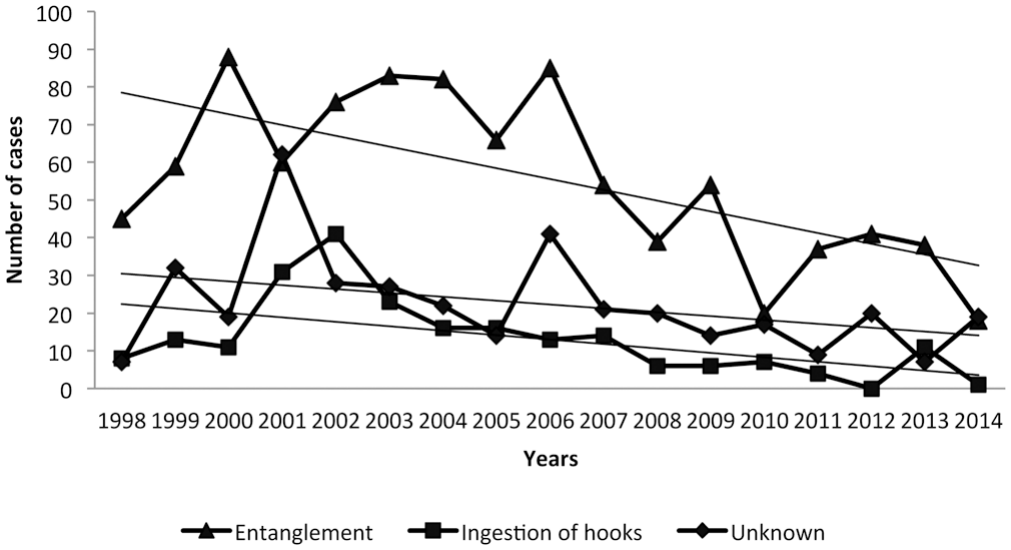
Tendency of the three most frequent causes of admissions during the period 1998–2014.

The total number of admissions peaked during 2001–2003 and since then decreased. Annual variation in causes of admission is shown in Fig 2. A significant decrease of cases was detected among the seventeen years of the study in all the stranding categories (Fig 3). Particularly interesting is the annual variation of cases of crude oil: 84.61% (n = 44) of crude oil admissions occurred during 1998–2005, detecting an important decrease since 2006.

### Final disposition

A total of 226 loggerhead turtles were dead when admitted. In these turtles, the most frequent causes of mortality were unknown/undetermined causes (70.35%, n = 159), entanglement (11.50%, n = 26), trauma (9.37%, n = 22), and ingestion of hooks (6.19%, n = 14).

The final disposition of the 1,634 loggerhead turtles admitted alive showed the following rates: E_r_ = 3.37% (n = 55), M_r_ = 10.34% (n = 169), R_r_ = 86.29% (n = 1,410). The final dispositions by causes of admission are shown in Table 2.

**Table 2:** Final disposition of the loggerhead turtles admitted alive during the period 1998–2014.

Cause of admission	Number of turtles	Final disposition					
		Euthanized		Dead		Released	
		Number	E_r_ (%)	Number	M_r_ (%)	Number	R_r_ (%)
**Entanglement**	919	21	2.28	49	5.33	849	92.38
**Hooks/monofilament lines**	207	7	3.38	36	17.39	164	79.22
**Trauma (boat strike)**	75	14	18.67	23	30.67	38	50.67
**Infectious disease**	102	6	5.88	26	25.49	70	68.62
**Crude oil**	49	1	2.04	2	4.08	46	93.87
**Other causes**	62	2	3.22	12	19.35	48	77.41
Ingestion of plastics	18	0	0	2	11.11	16	88.89
Buoyancy disorders	6	0	0	1	16.67	5	83.33
Shark attack	5	0	0	1	20	4	80
Malnutrition	24	0	0	8	33.33	16	66.67
Miscellany	9	2	22.22	0	0	7	77.78
**Unknown/undetermined**	220	4	1.81	21	9.54	195	88.63
**TOTAL**	**1,634**	**55**	**3.37**	**169**	**10.34**	**1,410**	**86.29**

**Fig 4:**
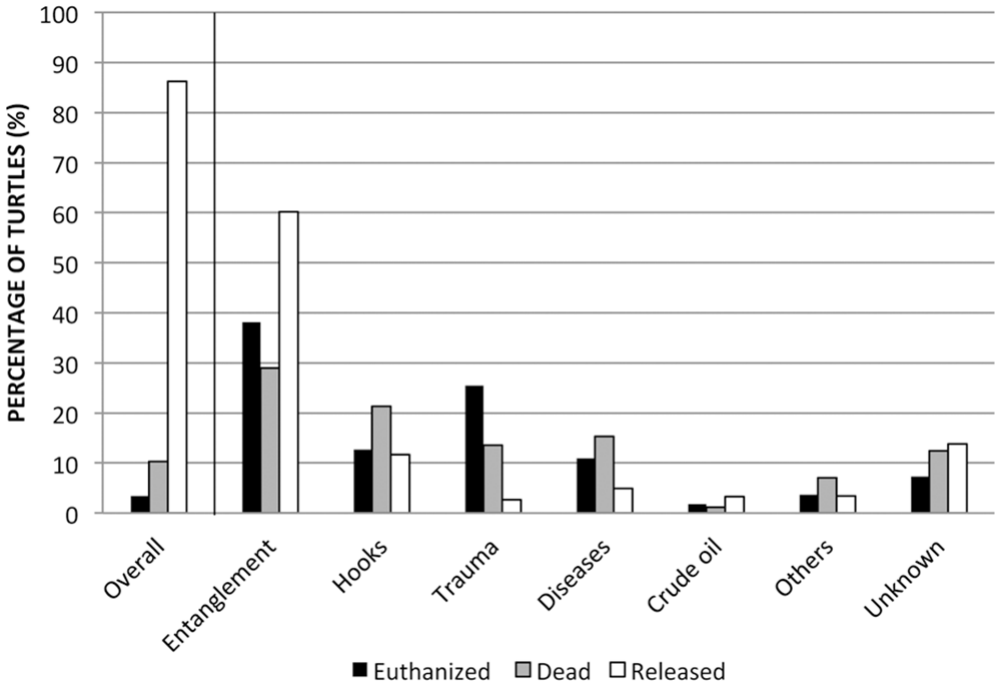
Resolution rates of euthanized (Er), dead (Mr), and released (Rr) loggerhead turtles relative to the overall population and the main cause of admission.

The euthanasia rate was significantly higher in the trauma category (18.67%) compared to the other main causes of admission (Fig 4). Turtles admitted due to trauma and infectious diseases had the highest unassisted mortality rates, 30.67% and 25.49%, respectively. The release rate was significantly higher in the crude oil (93.87%) and entanglement (92.38%) categories compared to the cause with the lowest release rate (trauma, 50.67%). In the subgroup of turtles with SCL known, only a significant difference was detected when release rates of pelagic juveniles and juvenile/subadult turtles for the unknown/undetermined category were compared (*P* = 0.021).

### Time until death and length of stay at the TWRC

Within the group of euthanized turtles the longest median T_d_ was observed for the unknown category (T_d_ = 85 days), whereas the shortest median T_d_ was recorded for the trauma category (T_d_ = 1 day) (Table 3). The median T_d_ in the dead turtles ranged from 1.5 days (crude oil) to 8 days (entanglement, trauma, and other causes). Within the group of released turtles the median time of stay in the TWRC ranged from 12 days (unknown) to 70 days (trauma).

**Table 3:** Statistical descriptive of time that loggerheads spent in the TWRC until the final disposition.

	Time (days) from admission to final disposition														
	Euthanasia					Unassisted mortality					Release				
CAUSE OF ADMISSION	P_10_	P_25_	P_50_	P_75_	P_90_	P_10_	P_25_	P_50_	P_75_	P_90_	P_10_	P_25_	P_50_	P_75_	P_90_
**Entanglement**	0	0	2	16.5	45.6	1	3	8	24	70	3	11	26	52	93
**Hooks**	0	0	3	61	70	0.6	1	3	13	30	7	19	38	67	151.1
**Trauma**	0	0	1	4.25	83	1	2	8	30	155.2	17.1	39.5	70	132	331.1
**Infectious disease**	2	4.25	26.5	44.7	47	0	1	4	16	32.7	12	19	43	63	132
**Crude oil**	N/A	N/A	N/A	N/A	N/A	1	1	1.5	2	2	2.8	9.5	16	29	52.4
**Other causes**	1	1	32.5	64	64	0.6	2	8	28.5	41.8	10.7	18.5	40	70.7	127
Ingestion of plastics	-		-	-	-	17	17	24.5	32	32	10	13	21.5	44	76.6
Buoyancy disorder	-		-	-	-	N/A	N/A	N/A	N/A	N/A	2	8	36	92	127
Shark attack	-		-	-	-	N/A	N/A	N/A	N/A	N/A	39	41.2	53.5	102.5	117
Malnutrition	-		-	-	-	0	2	2.5	18.7	46	18	35.2	43	112.7	154
Miscellany	1	1	32.5	64	64	-		-	-	-	2	14	56	118	270
**Unknown/ undetermined**	39	39	85	428	428	1	1	4	9.5	40	1	5	12	29	57

P_10_, P_25,_ P_50_, P_75_, P_90_: percentiles 10, 25, 50 (median), 75 and 90; N/A: not applicable (only one case)

## Discussion

Health status and anthropogenic threats of free-living sea turtle species are usually evaluated using as an important source of information the epidemiological studies of the causes of morbidity and mortality of turtles admitted to wildlife rehabilitation centers [9,14,21,26]. However, studies of the causes of morbidity of mortality of sea turtles covering more than one decade are scarce [14–16,21,29]. The present retrospective study included data of a long period (17 years), allowing a more accurate analysis of the annual variations and trends of the different causes of admission.

In our survey, the mean SCL of the stranded loggerheads was similar to that described for loggerheads from Madeira [34]. However, loggerheads from Azores have been reported to be smaller than those from Madeira, probably because they arrive first to Azores [35]. We found that the sex ratio in the group of turtles whose sex was determined was strongly female-biased (7/1). A 3.9/1 female-biased sex ratio was reported in a study conducted on 89 loggerheads stranded in Florida [26]. Loggerheads in the Canary Islands mainly come from the US western Atlantic by the Gulf Stream [3]. It has been described that three subpopulations (NE Florida, SE Florida, and Yucatán) produce jointly an estimated 75% female hatchlings [35]. However, laparoscopies as well as hormonal essays performed on loggerheads in Madeira indicated a female-biased sex ratio of 2/1 [35].

In our study, entanglement in fishing gear and/or plastics (synthetic raffia) was the main cause of admission (50.81%). Although entanglement has been reported as a common cause of morbidity and mortality in sea turtles [14,29,36,37], the prevalence of entanglement in the present study was higher than that reported in other surveys. Whereas the prevalence of entanglement in a retrospective study conducted on 3,861 sea turtles in Hawaii over a 22-year period was 5% [14], evidence of lesions caused by fishing gear was observed in 11.28% of the 5,938 turtles stranded in Italy during 1980–2008 [29]. The low prevalence of entanglement in Hawaii was also consequence of the high prevalence of fibropapillomatosis (28%) in the region [14]; however, no cases of fibropapillomatosis were reported in Italy [29]. Interactions with the activity of the canarian artisanal fishery are not very common; however, potential interactions between loggerheads and fisheries can take place in waters off Canary Islands mainly when “trasmallos” (a fishing net) are used to catch a wide range of fish species, from sharks and skates to many sparids, striped red mullet (*Mullus surmuletus*), etc., especially at the end of the summer [38]. Sometimes it is difficult to assess whether entanglement is the consequence of capture in fishing gear or of floating discarded fishing gear; in addition, entanglement in floating material like plastics is also an increasing problem [29]. In this way, it is remarkable than the use of synthetic raffia in the Canary Islands, particularly in the islands devoted to intensive agriculture, is very common. Ruminal disorders in goats due to ingestion of synthetic raffia have been reported in Gran Canaria Island [39]. Legal actions including the compulsory use of biodegradable raffia must be implemented in order to minimize its impact on sea turtle stranding in the Canary Islands.

Unknown/undetermined origin was the second category of stranding (20.37%) in our study. This prevalence was lower than the 49% reported for green turtle strandings in Hawaii [14]. Financial and time constraints usually make difficult to establish an accurate diagnosis in many stranded sea turtles [29]. In addition, rapid development of autolysis can impede more detailed examinations of the carcasses and the histological evaluation of the specimens [14,30]. There are also examples in which, although huge efforts were devoted to identify the cause of stranding, the specific cause could not be satisfactorily identified [23].

Ingestion of hooks and monofilament lines was the third cause of stranding (11.88%) in our survey. This prevalence was higher than that reported in green turtles stranded in Hawaii during 1982–2003 (7%) [14]. However, in a study on non-fibropapilloma causes of mortality in green turtles from Hawaii and the insular Pacifico, foreign body ingestion, including hooks and fishing lines, was observed in 12% of the turtles [16]. Estimated total take of sea turtles in the international waters in the North of the Canary Islands for 1983 to 1991 was 3,000 turtles [40]. However, more recently, impacts of fishing effort have been reported to be decreasing in the Canary Current because fish stocks are depleted throughout the region and management authorities are striving to reduce the fishing pressure [41]. Longline in waters off Canary Islands is deployed to catch benthopelagic to bathipelagic fish species, such as the european hake (*Merluccius merluccius*), the common mora (*Mora moro*) and even the meagre (*Argyrosomus regius*). All these species and other similar are caught with hooks, often baited [42]. In addition, other fishing fleets work in waters off Canary Islands using large longlines to catch sword fish (*Xiphias gladius*) [43]. Although many of the turtles are released by the fishermen, it has been estimated that approximately 15–50% of the turtles die due to the severe lesions induced by the fishing hooks [44]. Lesions induced by hooks and monofilament lines have been deeply described [15,16,30,45].

In our study, all the other primary causes had frequencies below 6%. As previously reported, it is remarkable the absence of fibropapillomatosis in the loggerheads stranded in the Canary Islands because sea turtle fibropapillomatosis is a disease of global distribution [30]. The prevalence of fibropapillomatosis in a retrospective study conducted on 3,861 stranded green turtles in Hawaii over a 22-year period was 28% [14]. Similarly, the prevalence of fibropapillomatosis in a retrospective survey conducted on 3,016 stranded green turtles in Florida during the period extending from 1980 to 1998 was 22.6% [21]. However, fibropapillomatosis was not present in other Pacific islands examined by Work et al. [16], and no cases of fibropapillomatosis were reported in a retrospective study conducted on 5,938 stranded loggerheads in Italy during 1980–2008 [29].

As previously reported in 2005 [30] no cases of spirorchiid infection were observed among loggerheads admitted to the TWRC during 1998–2014. Spirorchiid trematodes are implicated as an important cause of stranding and mortality in sea turtles worldwide [7,9,13,23]. In a retrospective study conducted on 100 stranded green turtles in Australia over a 4-year period, spirorchiidiasis was found to be the most frequently cause of mortality (41.8%) [9]. However, although high prevalence of spirorchiid infection was observed in a survey conducted on 148 sea turtles in Florida, most infections were regarded as incidental to the cause of death [26]. A high percentage of green turtles with fibropapillomatosis in Hawaii are also infected with spirorchiid trematodes [10,13]. Detection of infection with spirorchiids in turtles usually is done at necropsy, when adult worms or eggs are observed either grossly or at microscopy [13]. None of the necropsied loggerheads included in our study had intravascular adult flukes or trematode eggs in tissues. Antemortem detection of infection is also possible using enzyme-linked immunosorbent assays [13,46,47]. However, no antemortem diagnosis using serology was attempted on the loggerheads included in our study due to financial constraint. Work et al. [13] hypothesized that immature green turtles become infected with spirorchiids shortly after recruiting into coastal foraging pastures from the pelagic environment. All necropsied loggerheads included in our survey were juvenile and subadult specimens but different alimentary habits of both species can explain the absence of spirorchiid fluke infection. Sea turtles acquire the flukes by ingesting unknown cercaria-rich intermediate hosts. Although preliminary results on detection of spirorchiids in gastropod tissues by polymerase chain detection have been reported [48], the only life cycles that have been established in this group were for species in the freshwater genera *Enterohaemototrema*, *Spirorchis*, and *Vasotrema* [49].

Because the Canary Islands are included throughout the year in a thermal gradient centered on the 21°C isotherm [50], no cases of cold-stunning were observed among loggerheads included in our study. However, hypothermic stunning events have been described affecting high number of sea turtles in Florida [20,24].

In our survey, seasonal analysis of the strandings showed that these were more frequent in summer, probably reflecting loggerheads are more abundant around the Canary Islands in this season. It has also been reported that loggerheads are more abundant in Madeiran waters during the summer months [35]. In addition, the total number of admissions included in our study peaked during 2001–2003 and since then decreased. Several studies have indicated that the abundance of loggerhead nests along the Atlantic coast and in southwestern Florida is declining [51,52]. Because loggerheads in the Canary Islands mainly come from the US western Atlantic, decline in nest abundance on those beaches could have a negative impact on the juvenile and subadult loggerhead population around the Canary Islands. Skeletochronology of mostly loggerheads from Madeira showed the duration of the oceanic stage to be equal or longer than 7 years [53]. No studies on determination of the age of the loggerheads around the Canary Islands have been published. Assuming this age is similar to that reported for Madeiran loggerheads, the decrease observed in our study, particularly since 2003, could be consequence of the decrease of nests on the US western Atlantic coast since mid 90’s. Estimated declines in nest abundance on the Atlantic and southwestern coasts of Florida ranged from 29% to 37% between 1989 and 2006 [52].

It is remarkable that admissions due to crude oil decreased significantly since 2006. In a previous study on crude oil as stranding cause among loggerheads in the Canary Islands, authors concluded that the designation of the Canary Islands as a Particularly Sensitive Sea Area (PSSA) in 2005 by the International Maritime Organization (IMO) was associated with positive effects on the reduction of sea turtle strandings caused by crude oil [54]. A PSSA is an area of the marine environment that needs special protection through action by the IMO because of its significance for recognized ecological, socio-economic, or scientific attributes where such attributes may be vulnerable to damage by international shipping activities [55]. The associated protective measures after the international recognition of the waters of the Canary Islands as PSSA were adopted in 2006, and included: traffic separation systems (recommended routes), areas to be avoided, and mandatory ship reporting system [55].

Wildlife clinical practice guidelines dealing with welfare rehabilitation standards and pre-release health screening protocols have been published [56,57], but no quality indicators of the rehabilitation process of injured sea turtles have been defined. In our study, we analyzed the outcomes of the rehabilitation of free-living loggerhead turtles at the TWRC, adopting the three categories of the final disposition, the time until death and the length of stay, as indicators of the quality audit of the rehabilitation process before release into the wild.

According to our data, 86.29% of loggerheads admitted alive to the TWRC were successfully released, and only 13.71% of turtle admissions resulted in euthanasia or unassisted mortality. References on the final dispositions of sea turtle rehabilitation are scarce because they usually have been focused on the causes of mortality [14].

Based on animal welfare, euthanasia is a final option in all wildlife species rehabilitation [58]. In our survey, the overall rate of euthanasia was 3.37%, and the highest value was found in the trauma (boat strikes) category (18.67%). Turtles with boat strike injuries usually have severe fractures of the carapace/plastron, and severe traumatic lesions mainly penetrating into the lungs and kidneys [30]. Affection of these vital organs, because the anatomical location, dorsally attached to the carapace, explain the generally poor prognosis for turtles with severe traumatic injuries in the carapace [30].

Unassisted mortality rate has been used as a quality indicator parameter in rehabilitation of birds of prey [59]; however, no reports on quality auditing of the rehabilitation process in free-living sea turtles admitted at marine rehabilitation centers have been published. In our study, the overall rate of unassisted mortality was 10.34%, and the highest value was found in the trauma (boat strikes) category (30.67%). The reasons for the poor prognosis for these turtles have been explained above. A high M_r_ (25.49%) was also found in the infectious disease category. According to the necropsy findings and microbiological studies, septicemia was diagnosed in the majority of those cases [30].

The release rate in our survey was higher in the crude oil (93.87%) and entanglement (92.38%) categories compared to the cause with the lowest release rate (trauma, 50.67%). Ingestion of crude oil and subsequent internal lesions can threaten sea turtle survival, but lesions in the skin, carapace, and plastron are not fatal in the majority of cases [53]. Entanglement in fishing gear and/or plastics can result in severe ulcerative dermatitis, and amputation of flippers [30]; however, sea turtles cope with amputations well, regardless of whether the amputation is front or rear [60].

The parameter time to death provides direct insight into the initial assessment and prognostication, the overall rehabilitation process, and the validity of veterinary protocols [59]. In our study, the shortest median time to euthanasia was recorded for the trauma category (1 day) meaning that the decision is made very soon based on the poor prognosis of these cases as was discussed above. Within the group of dead turtles during the rehabilitation process, the median time to death ranged from 1.5 days (crude oil) to 8 days (entanglement, trauma, and other causes). This fact suggests that first week of stay at the rehabilitation center is critical, and intensive cares should be performed on all turtles during the first week, despite their apparently less severe appearance.

The parameter length of stay must be as short as possible to reduce the risk of captive-related complications, infectious diseases, and behavioral disorders [61]. In our study, the median time of stay in the TWRC ranged from 12 days (unknown) to 70 days (trauma). This fact suggests that especially turtles admitted due to trauma represent an important consume of time and efforts.

In conclusion, this survey is the first large-scale epidemiological study on causes of stranding and mortality of Eastern Atlantic loggerhead sea turtles, providing useful information for the conservation of these reptiles. In absence of diseases commonly reported in other regions, such as fibropapillomatosis [14,21] and spirorchiidiasis [7,9,13,23,26], at least 71.72% (n = 1,334) of loggerheads included in our study stranded due to anthropogenic causes, what deserves critical reflection, especially taking into account that stranded loggerhead turtles may represent as little as 7% of the at-sea mortality of sea turtles [62]. In addition, the high survival rate for stranded loggerheads (86.29%) achieved at the TWRC emphasizes the importance of marine rehabilitation centers for the conservation of sea turtles. Finally, we propose that at least the stratified analysis by causes of admission of the three final disposition rates (E_r_, M_r_, and R_r_), and the parameters time until death (T_d_) and length of stay at the center (T_r_) should be included in the outcome research of the rehabilitation process of free-living sea turtles in order to allow comparative studies between marine rehabilitation centers around the world.

## Supporting Information

S1 File Data of stranded loggerhead turtles admitted at the TWRC during the period 1998–2014. (XLSX)
